# Overexpression of ATP sulfurylase improves the sulfur amino acid content, enhances the accumulation of Bowman–Birk protease inhibitor and suppresses the accumulation of the β-subunit of β-conglycinin in soybean seeds

**DOI:** 10.1038/s41598-020-72134-z

**Published:** 2020-09-14

**Authors:** Won-Seok Kim, Jeong Sun-Hyung, Nathan W. Oehrle, Joseph M. Jez, Hari B. Krishnan

**Affiliations:** 1 Plant Science Division, University of Missouri, Columbia, MO 65211 USA; 2 Plant Genetics Research, USDA-Agricultural Research Service, University of Missouri, 108 Curtis Hall, Columbia, MO 65211 USA; 3 Department of Biology, Washington University in St. Louis, St. Louis, MO 63130 USA

**Keywords:** Biotechnology, Plant sciences

## Abstract

ATP sulfurylase, an enzyme which catalyzes the conversion of sulfate to adenosine 5′-phosphosulfate (APS), plays a significant role in controlling sulfur metabolism in plants. In this study, we have expressed soybean plastid ATP sulfurylase isoform 1 in transgenic soybean without its transit peptide under the control of the 35S CaMV promoter. Subcellular fractionation and immunoblot analysis revealed that ATP sulfurylase isoform 1 was predominantly expressed in the cell cytoplasm. Compared with that of untransformed plants, the ATP sulfurylase activity was about 2.5-fold higher in developing seeds. High-resolution 2-D gel electrophoresis and immunoblot analyses revealed that transgenic soybean seeds overexpressing ATP sulfurylase accumulated very low levels of the β-subunit of β-conglycinin. In contrast, the accumulation of the cysteine-rich Bowman–Birk protease inhibitor was several fold higher in transgenic soybean plants when compared to the non-transgenic wild-type seeds. The overall protein content of the transgenic seeds was lowered by about 3% when compared to the wild-type seeds. Metabolite profiling by LC–MS and GC–MS quantified 124 seed metabolites out of which 84 were present in higher amounts and 40 were present in lower amounts in ATP sulfurylase overexpressing seeds compared to the wild-type seeds. Sulfate, cysteine, and some sulfur-containing secondary metabolites accumulated in higher amounts in ATP sulfurylase transgenic seeds. Additionally, ATP sulfurylase overexpressing seeds contained significantly higher amounts of phospholipids, lysophospholipids, diacylglycerols, sterols, and sulfolipids. Importantly, over expression of ATP sulfurylase resulted in 37–52% and 15–19% increases in the protein-bound cysteine and methionine content of transgenic seeds, respectively. Our results demonstrate that manipulating the expression levels of key sulfur assimilatory enzymes could be exploited to improve the nutritive value of soybean seeds.

## Introduction

Soybean is an economically important crop that serves as a major protein source for both animal feed and human food throughout the world. The United States is a leading producer of soybean and produced roughly 123.7 million metric tons of soybeans in 2018 alone (https://soygrowers.com/wp-content/uploads/2019/10/Soy-Stats-2019_FNL-Web.pdf). A significant portion of soybean meal is used in animal feed with poultry and swine being the main consumers. Soybean meal is sought after in the feed industry because it is considered as the “gold standard” when compared with other protein sources. Soybean has become indispensable in animal feed because of its high protein content, protein digestibility, well-balanced amino-acid profile, availability and relative cost to produce.

Though soybeans are an excellent protein source, its quality could be further enhanced if the sulfur amino acid content of soybeans could be elevated^1,2^. Methionine and cysteine are the two sulfur-containing amino acids in plants^3^. Methionine is considered as an “essential” amino acid because humans and monogastric animals cannot synthesize this metabolite and are dependent on obtaining it through food /feed that is being consumed. Cysteine is considered as a “conditionally” essential amino acid since animals can convert methionine consumed with their diet into cysteine. Deficiency in these sulfur-containing amino acids has a negative influence on the growth and development of animals^4^. Thus, improving the concentration of these two sulfur-containing amino acids will greatly enhance the nutritive value of soybeans.

Biotechnological approaches have been undertaken to elevate the sulfur-amino acid content of legume seeds^2,5–7^. One common approach has been to overexpress methionine-rich heterologous proteins in transgenic plants^2^. Sulfur-rich zeins from maize, 2S albumin from Brazil nut and sunflower have been expressed in transgenic soybeans. Expression of these proteins have resulted in modest increase in the overall sulfur amino acid content of soybeans; however, the overall improvement of the sulfur-amino acid content has not been satisfactory to meet the poultry and livestock requirements. One major constraint in this approach has been insufficient accumulation of methionine-rich proteins in soybean seeds. Additionally, expression of heterologous proteins often resulted in reduced accumulation of endogenous sulfur-rich proteins. This observation indicates that there is a limitation in the availability of methionine and cysteine in soybean seeds.

Recent studies have overexpressed enzymes involved in the sulfur assimilatory and cysteine/methionine biosynthesis pathways to improve the sulfur amino acid content of seeds^8–14^. Overexpression of a cytosolic isoform of O-acetylserine sulfhydrylase (OASS) resulted in a 58–74% increase in the protein-bound cysteine in transgenic soybeans when compared to the wild-type plants^9^. Transgenic soybeans overexpressing Arabidopsis cystathionine γ-synthase resulted in 2.3-fold increase in methionine content of the seed^10–12^. These examples indicate that manipulation of sulfur assimilatory and/or methionine biosynthetic enzymes may be a viable option to increase the sulfur-amino acid content of soybean seeds.

Plants take up sulfur from the soil in the form of sulfate, a process mediated by sulfur transporters. Once inside the plant, the sulfate is activated to adenosine 5′-phosphosulfate (APS) a reaction catalyzed by ATP sulfurylase (ATP:sulfate adenylyltransferase; EC 2.7.7.4). Production of APS by ATP sulfurylase is the committed step in plant sulfur assimilation and yields a high energy phosphosulfate bond that drives subsequent metabolic steps in the pathway^15–17^. ATP sulfurylase enzymes are encoded by multigene families in plant species and are localized in different organelles^18–23^. Earlier, we cloned and characterized ATP sulfurylase from soybean^23^. Soybean contains four ATP sulfurylase isoforms (GmATP sulfurylase1–4) that are abundantly expressed in young leaves, young seeds, and the elongation zone of roots^23^. Soybean ATP sulfurylase isoform 1 (GmATP sulfurylase) is a dimeric protein of ∼100 kDa formed from two 48-kDa monomers^23–25^. The x-ray crystal structure of soybean ATP sulfurylase 1 in complex with APS has been elucidated resulting in a better understanding on how this enzyme functions as the committed step in plant sulfur assimilation^25^. Since ATP sulfurylase contributes significantly to the control of plant sulfur metabolism, we anticipated that overexpression of this enzyme may lead to increased synthesis of sulfur amino acids. To examine this possibility, we generated transgenic soybean plants that constitutively overexpress soybean ATP sulfurylase 1 without its transit peptide. Soybean seeds overexpressing ATP sulfurylase revealed altered seed protein composition with preferential accumulation of cysteine-rich Bowman–Birk protease inhibitor (BBI). Importantly, the sulfur amino acid content of ATP sulfurylase overexpressing soybean seeds was significantly improved.

## Results

### Generation of transgenic soybean plants overexpressing ATP sulfurylase

**Figure 1:**
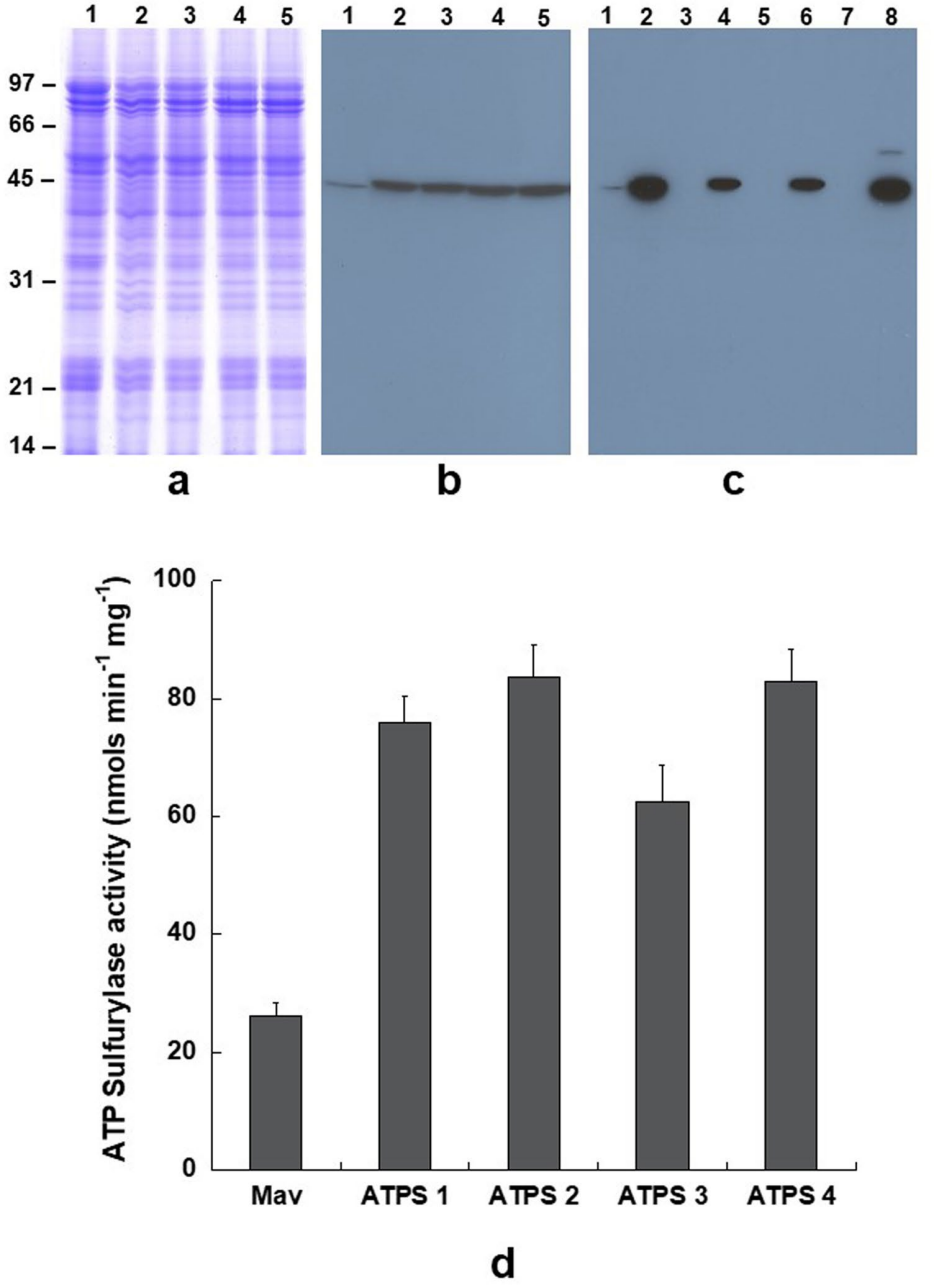
Immunological detection of ATP sulfurylase in developing seeds of transgenic soybean and non-transformed control plants. Total seed proteins from non-transformed control (lane 1) and four independent transgenic lines (lanes 2–5) were fractionated by 13.5% SDS-PAGE and stained with Coomassie Blue (Panel **a**) or transferred to a nitrocellulose membrane and probed with antiserum against soybean ATP sulfurylase (Panel **b**). Proteins reacting against the antibodies were identified using anti rabbit IgG-horseradish peroxidase conjugate followed by chemiluminescent detection. Lane 1, non-transformed control; lane 2, ATPS1; lane 3, ATPS2; lane 4, ATPS3, and lane 5, ATPS4. (Panel **c**) Detection of ATP sulfurylase in different subcellular fractions. Total leaf protein from Maverick and ATPS sulfurylase (lanes 1and 2) were subjected to differential centrifugation and the resulting subcellular fractions (chloroplast lanes 3 and 4), endoplasmic and plasma membrane (lanes 5 and 6) and cytosol (lanes 7 and 8) were separated by SDS-PAGE and immunoblot analysis. (Panel **d**) ATP sulfurylase activity in seed extracts of wild-type and four independent transgenic soybean lines. ATP sulfurylase activity from wild-type and transgenic soybeans are presented as the means ± SE (n = 3).

A plant transformation construct harboring the coding region of soybean ATP sulfurylase 1 without the transit peptide under control of the CaMV 35S promoter was inserted into genome of soybean cultivar Maverick by *Agrobacterium*-cotyledonary node transformation. This procedure resulted in the regeneration of four independent transgenic events (ATPS1, ATPS2, ATPS3, and ATPS4) that showed resistance to Liberty herbicide. To verify the expression of ATP sulfurylase in these transgenic plants, immunoblot analysis was performed with antibodies raised against soybean ATP sulfurylase. Total protein obtained from young seeds (20 days after flowering) of four independent transgenic soybean lines and a non-transgenic soybean line were resolved by SDS-PAGE (Fig. 1a). Immunoblot results showed strong reaction against a 42 kDa protein from all the four independent transgenic soybean lines while only a faint reaction was detected in the non-transgenic control soybean line (Fig. 1b). To verify the distribution of the introduced ATP sulfurylase, which was expressed in the absence of a transit peptide, we isolated different crude subcellar fractionations by differential centrifugation. Immunoblot analysis revealed ATP sulfurylase was predominantly distributed in the cytosol, although lower amounts were also found in other fractions (Fig. 1c). We also measured the ATP sulfurylase activity in these transgenic soybean lines. When compared to the non-transgenic control soybean line, ATP sulfurylase activity was 2 to 2.5-fold higher in the transgenic soybean lines (Fig. 1d).

### Overexpression of ATP sulfurylase alters seed protein content and composition

To investigate if overexpression of ATP sulfurylase brought about changes in seed components, we measured the total protein and oil content of field grown mature dry seeds. The total protein content of seeds overexpressing ATP sulfurylase (37.1 ± 0.5%) was about 3% lower than that observed in non-transgenic control seeds (40.1 ± 0.6%). In contrast, the oil content of the ATP sulfurylase overexpressing seeds (22.3 ± 0.3%) was about 1% higher than the wild-type seeds (21.1 ± 0.4%). We also observed differences in seed yield, though statistically non-significant, between ATP sulfurylase overexpressing plants (52.46 g ± 11.86 g/plant) and the control plants (59.78 g ± 14.30 g/plant). Similarly, a decrease in seed weight was observed in ATP sulfurylase overexpressing plants (13.96 g ± 0.74 g/100 seeds) when compared to the non-transgenic control seeds (16.62 g ± 1.03 g/100 seeds).

**Figure 2:**
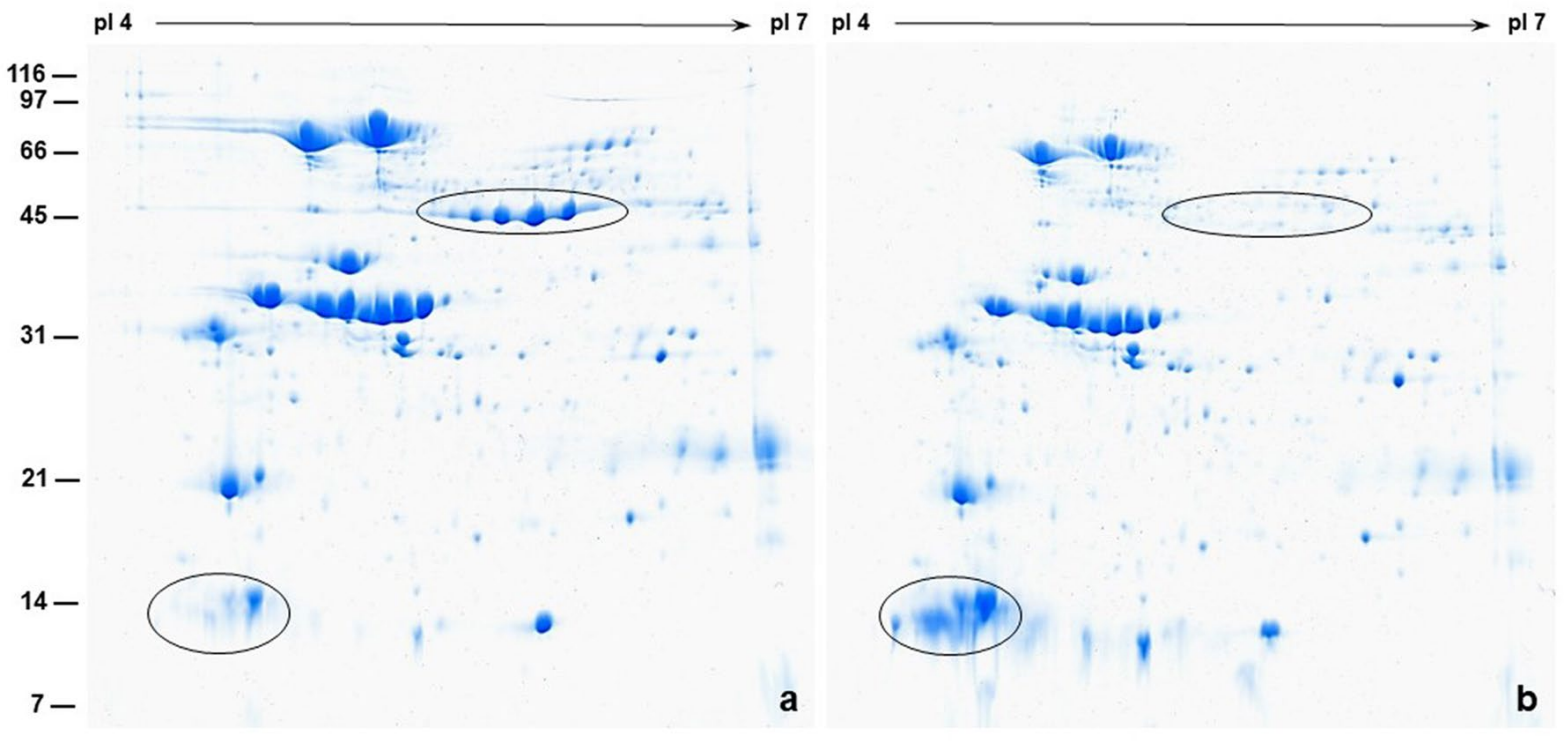
High-resolution two-dimensional gel electrophoresis comparison of seed proteins. Seed proteins (300 μg) from wild type control (Panel **a**) and ATP sulfurylase overexpressing transgenic seeds (Panel **b**) were first separated by isoelectric focusing on pH 4–7 strips followed by separation by SDS-PAGE on 15% gels. The gels were stained with Colloidal Coomassie Blue G-250. Protein spots corresponding to the β-subunit of β-conglycinin and the Bowman-Birk protease inhibitors are circled. The position and sizes of the molecular weight markers in kDa are shown on the left side of the figure.

The decrease of protein content in ATP sulfurylase overexpressing mature seeds prompted us to investigate if this loss was accompanied by changes in the protein composition. For this purpose, we conducted high-resolution 2-D gel electrophoresis of mature soybean seed proteins (Fig. 2). Examination of Coomassie stained 2-D gels revealed several discrete protein spots. We and others have previously identified these 2-D resolved proteins by mass spectrometry^26–28^. The two most abundant soybean seed storage proteins are the 7S β-conglycinin and 11S glycinin, respectively. In the wild-type seeds, the three sub-units of β-conglycinin migrated as abundant proteins spots with apparent molecular weight of 76 kDa, 72 kDa, and 52 kDa, respectively (Fig. 2a). The β-subunit of β-conglycinin migrated as five distinct protein spots with slightly different isoelectric points (Fig. 2a). Interestingly, the abundance of these protein spots in ATP sulfurylase overexpressing soybean seeds was drastically reduced compared to the wild-type seeds (Fig. 2b). The other abundant seed storage protein, the glycinin which is made of acidic and basic subunits, resolved into discrete protein spots migrating with apparent molecular weights of 32–34 kDa and 20–22 kDa, respectively (Fig. 2). The abundance of these protein spots was determined by Delta2D image analysis (Supplemental Fig. 1 and Supplemental Table 1). The percent spot volume intensity of some the abundant seed protein spots were relatively higher in ATP sulfurylase overexpressing soybean compared to wild-type plants (Supplemental Table 1). Additionally, several low molecular weight protein spots migrating at 12–14 kDa also accumulated at higher amounts in the ATP sulfurylase overexpressing soybean seeds (Supplemental Table 1).

**Table 1:** Amino acid composition of dry seeds of Maverick and ATP sulfurylase overexpressing soybean plants.

	Maverick	ATPS-1	ATPS-2	ATPS-3	ATPS-4
**Essential amino acid**					
Thr	4.66 ± 0.12^A^	4.75 ± 0.09^A^	4.80 ± 0.05^A^	4.70 ± 0.06^A^	4.76 ± 0.08^A^
Val	5.75 ± 0.02^A^	5.59 ± 0.06^B^	5.59 ± 0.07^B^	5.49 ± 0.06^BC^	5.45 ± 0.05^C^
MetSO_2_	1.47 ± 0.06^B^	1.71 ± 0.04^A^	1.69 ± 0.04^A^	1.69 ± 0.06^A^	1.75 ± 0.06^A^
Ile	4.91 ± 0.01^A^	4.89 ± 0.05^AB^	4.90 ± 0.04^A^	4.89 ± 0.05^AB^	4.82 ± 0.06^A^
Leu	7.54 ± 0.03^A^	7.42 ± 0.05^AB^	7.45 ± 0.08^AB^	7.42 ± 0.05^AB^	7.33 ± 0.05^C^
Phe	4.15 ± 0.05^A^	3.92 ± 0.17^A^	4.06 ± 0.27^A^	4.02 ± 0.27^A^	3.94 ± 0.28^A^
Lys	5.28 ± 0.16^A^	5.66 ± 0.26^A^	5.40 ± 0.51^A^	5.46 ± 0.39^A^	5.51 ± 0.42^A^
**Nonessential amino acid**					
Asp	10.21 ± 0.24^A^	10.31 ± 0.46^A^	10.13 ± 0.31^A^	10.37 ± 0.46^A^	10.61 ± 0.78^A^
Ser	6.65 ± 0.11^A^	6.75 ± 0.07^A^	6.77 ± 0.18^A^	6.81 ± 0.09^A^	6.80 ± 0.13^A^
Glu	14.72 ± 0.25^A^	14.30 ± 0.42^A^	14.19 ± 0.24^A^	14.39 ± 0.53^A^	14.43 ± 0.84^A^
Gly	8.51 ± 0.10^A^	8.36 ± 0.17^A^	8.58 ± 0.20^A^	8.54 ± 0.21^A^	8.31 ± 0.60^A^
Ala	5.93 ± 0.05^A^	6.03 ± 0.19^A^	5.89 ± 0.29^A^	5.84 ± 0.18^A^	5.92 ± 0.19^A^
Cya	1.83 ± 0.04^C^	2.58 ± 0.05^AB^	2.51 ± 0.13^A^	2.61 ± 0.14^AB^	2.78 ± 0.16^A^
Tyr	3.12 ± 0.06^A^	3.06 ± 0.10^A^	3.15 ± 0.19^A^	3.14 ± 0.18^A^	3.12 ± 0.21^A^
His	2.55 ± 0.05^A^	2.56 ± 0.10^A^	2.62 ± 0.14^A^	2.59 ± 0.16^A^	2.58 ± 0.18^A^
Arg	6.52 ± 0.11^A^	5.92 ± 0.25^AB^	6.04 ± 0.19^AB^	5.84 ± 0.28^B^	5.79 ± 0.29^B^
Pro	6.19 ± 0.02^A^	6.18 ± 0.12^A^	6.23 ± 0.05^A^	6.18 ± 0.07^A^	6.11 ± 0.10^A^
*T.S.A.A	3.30 ± 0.10	4.29 ± 0.09	4.20 ± 0.17	4.30 ± 0.20	4.53 ± 0.22

Data is expressed as mole % ± standard error. Values followed by the same letter are not significantly different.

*total sulfur amino acids (cysteine + methionine).

### Immunoblot analysis confirms overexpression of ATP sulfurylase suppresses the accumulation of the β-subunit of β-conglycinin and promotes the accumulation of Bowman–Birk protease inhibitor (BBI)

**Figure 3:**
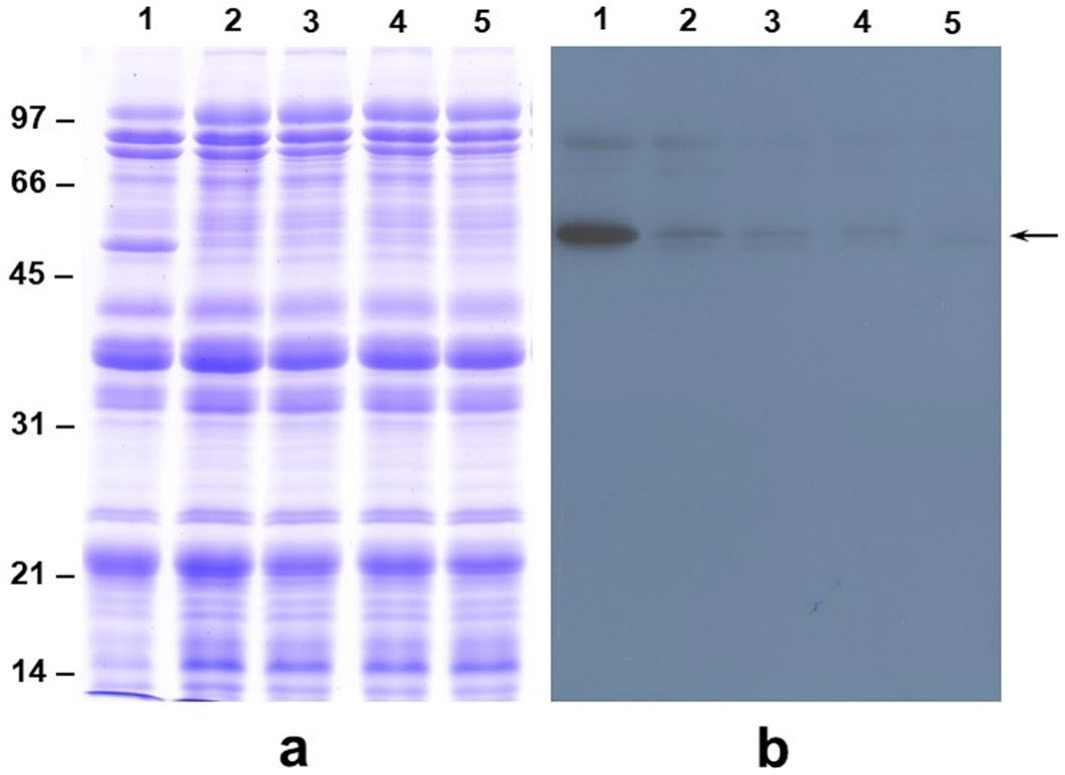
Total protein profile of mature dry seeds of non-transformed wild-type and transgenic soybean plants. Total seed proteins from non-transformed control (lane 1) and four independent transgenic lines (lanes 2–5) were fractionated by 12% SDS-PAGE and stained with Coomassie Blue (Panel a) or transferred to a nitrocellulose membrane and probed with antiserum raised against the β-subunit of β-conglycinin antibody (Panel b). Note the drastic reduction in the accumulation of the β-subunit of β-conglycinin. Lane 1, non-transformed control; lane 2, ATPS1; lane 3, ATPS2; lane 4, ATPS3, and lane 5, ATPS4.

**Figure 4:**
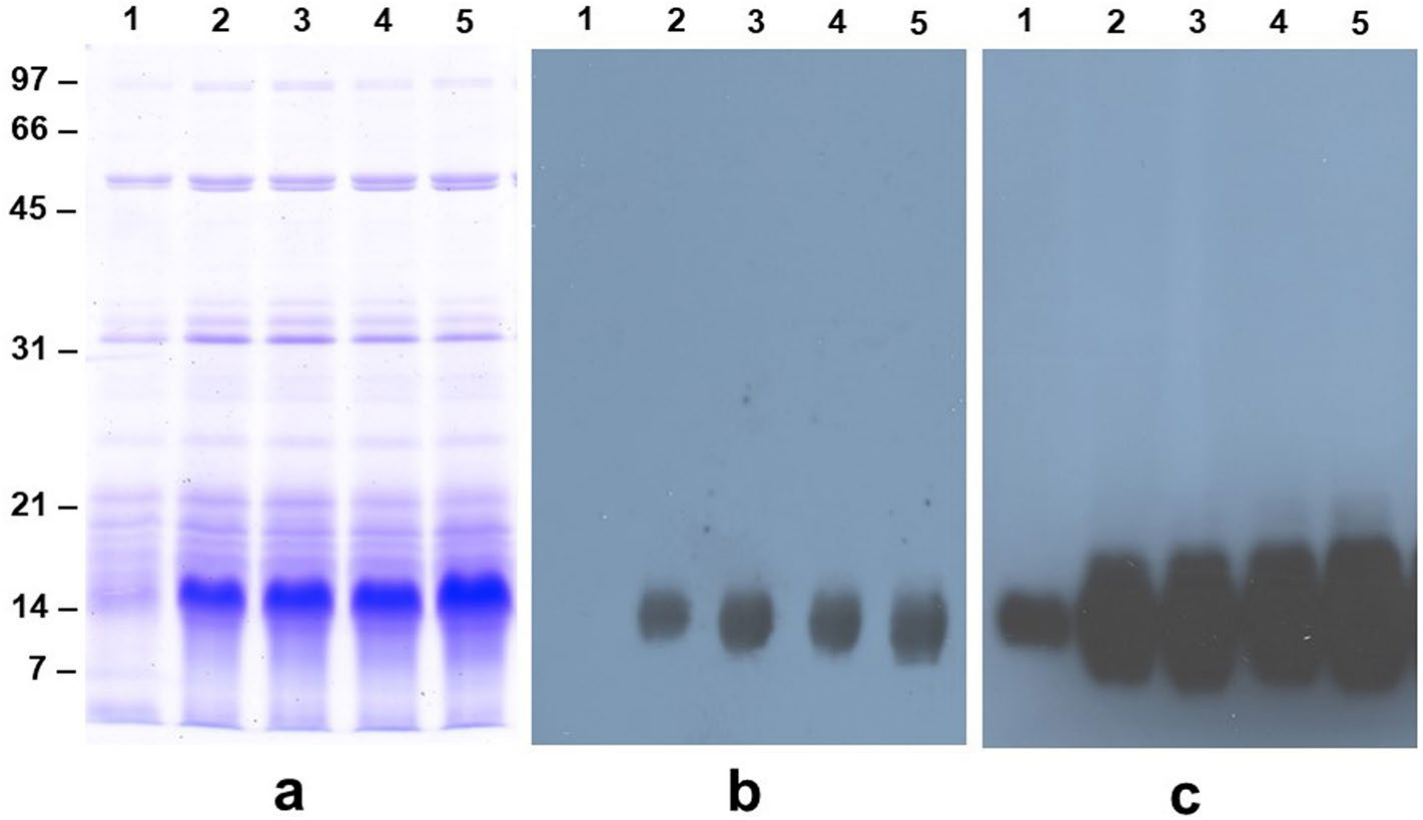
Immunoblot detection of Bowman–Birk protease inhibitor in the seeds of non-transformed wild-type and transgenic soybean plants. Panel a shows Coomassie Blue R-250 stained 50%-isopropanol extracted seed proteins that were separated by 13.5% SDS-PAGE. Panel b and c are an immunoblot analysis using antiserum raised against Bowman-Birk protease inhibitor. Panel b was exposed for 2 s while Panel c was exposed for 2 min. Lane 1, non-transformed control; lane 2, ATPS1; lane 3, ATPS2; lane 4, ATPS3, and lane 5, ATPS4.

Our high-resolution 2-D gel analysis indicated that overexpressing ATP sulfurylase in transgenic soybean leads to major alterations in the seed protein profiles (Fig. 2 and Supplemental Table 1). To confirm these changes and verify the identity of these proteins we performed immunoblot analysis using polyclonal antibodies raised against the β-subunit of β-conglycinin (Fig. 3) and BBI (Fig. 4). SDS-PAGE analysis of the total seed proteins from the dry seeds of four independent transgenic plants clearly showed the absence of a 52 kDa protein (Fig. 3a). In contrast, this protein was prominently visible in the wild-type seed protein extracts. Antibodies raised against the β-subunit of β-conglycinin reacted strongly against this polypeptide, while only a faint reaction can be detected in the transgenic soybean seeds (Fig. 3b).

Since our 2-D gel comparison indicated that the BBI accumulation may be enhanced in the ATP sulfurylase overexpressing soybean seeds, we conducted immunoblot analysis to confirm this observation. Previously, we demonstrated that 50% isopropanol can be used to preferentially extract BBI from soybean seeds^29,30^. Therefore, we extracted 50% isopropanol-soluble proteins from the dry seeds of four independent transgenic plants. SDS-PAGE analysis of 50% isopropanol-soluble proteins revealed similar protein profile between the control and transgenic plants (Fig. 4). A group of proteins migrating with apparent molecular weights of 10–14 kDa were much more abundant in the transgenic seeds (Fig. 4a). Antibodies raised against soybean BBI reacted strongly against the 10–14 kDa polypeptides confirming that these proteins are isoforms of BBI (Fig. 4b). In contrast, a longer exposure was required to detect BBI accumulation in wild-type seeds clearly establishing that BBI accumulates several-fold higher in the transgenic soybean seeds compared to non-transgenic seeds (Fig. 4c).

### Impact of overexpression of ATP sulfurylase on seed metabolites

**Figure 5:**
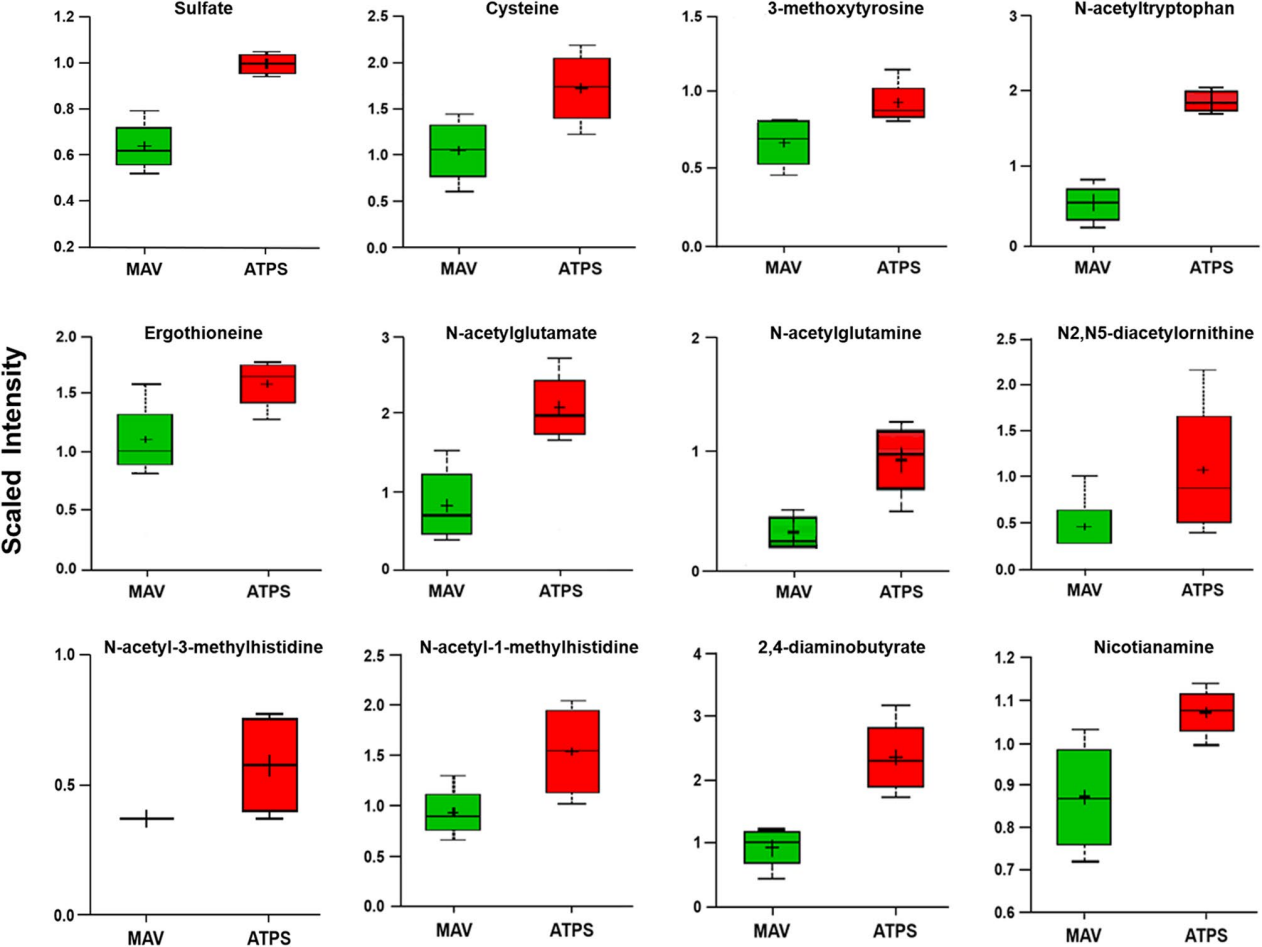
Metabolites elevated in ATP sulfurylase overexpressing transgenic seeds. Box plots were generated for metabolites that showed significant increase using both t-test and false discovery rate (FDR), with *p* < 0.05 and q < 0.10 as significant levels. Mav refers to wild-type seeds and ATPS refers to ATP sulfurylase overexpressing transgenic seeds. The numbers on the y-axis refer to relative abundance of metabolites.

**Figure 6:**
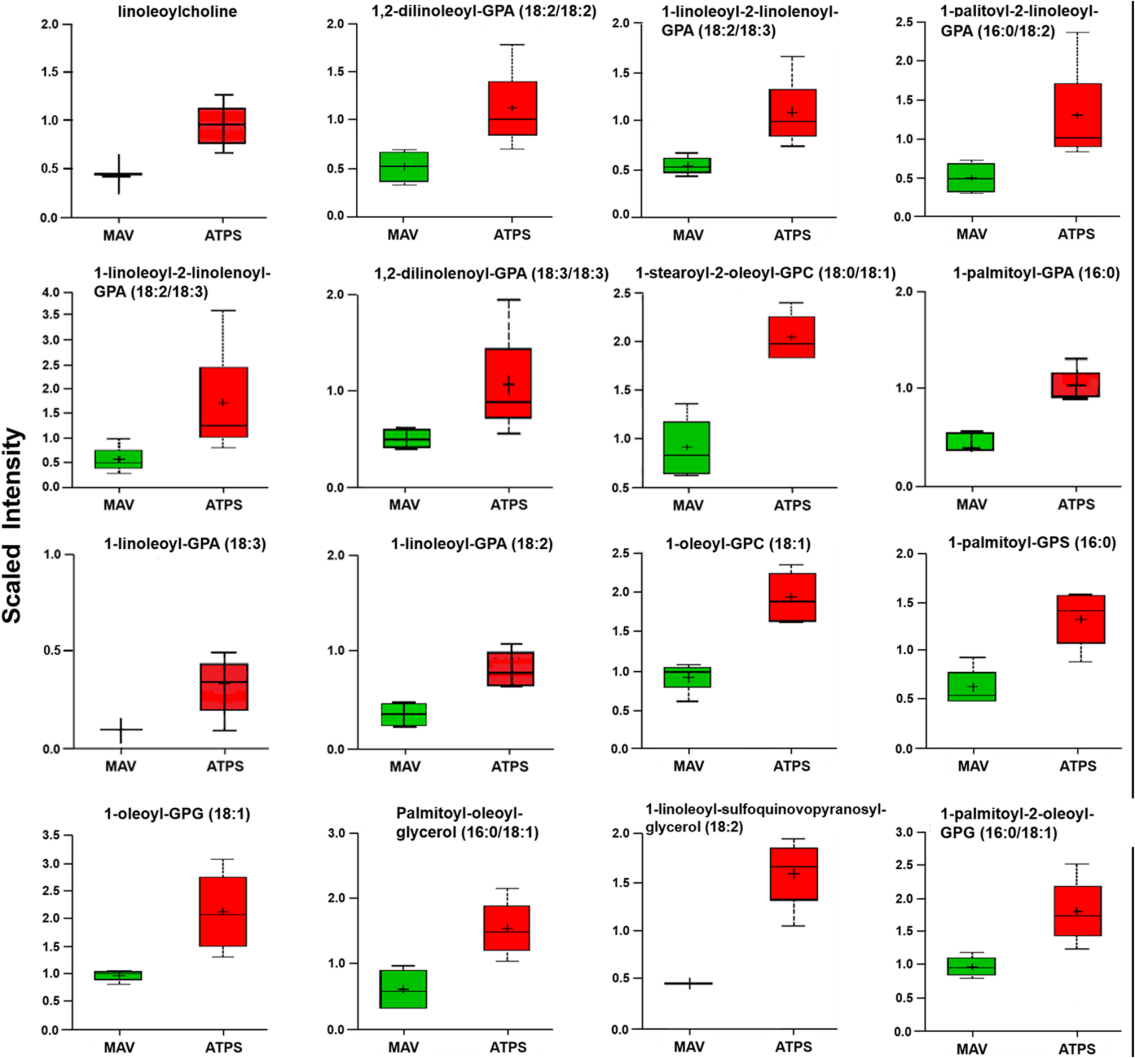
Graphical representation of several classes of lipids in wild type (MAV) and ATP sulfurylase (ATPS) overexpressing transgenic seeds. Box plots were generated for metabolites that showed significant increase using both t-test and false discovery rate (FDR), with *p* < 0.05 and q < 0.10 as significant levels. The numbers on the y-axis refer to relative abundance of metabolites.

To obtain insight into the biochemical changes resulting from overexpression of ATP sulfurylase, we carried out metabolite profiling of soybean seeds. By employing a combination of metabolite profiling platforms a total of 124 metabolite compounds were detected, quantified and analyzed (Supplemental Table 2). A heat map of metabolite levels in dry seeds of wild-type and ATP sulfurylase overexpressing soybean plants showed significant differences in several metabolites belonging to major biochemical pathways (Supplemental Fig. 3). This includes 38 amino acids and amino acid derivatives, 9 carbohydrates, 63 lipids, 6 cofactors/prosthetic groups/electron carriers, 3 nucleotides, 4 secondary metabolites, and 1 xenobiotic chemical. Out of 124 metabolites analyzed, 84 were increased and 40 decreased in ATP sulfurylase overexpressing seeds compared to non-transformed wild-type seeds (Supplemental Table 2). Figure 5 shows box plot layouts of several metabolites that are significantly elevated in ATP sulfurylase overexpressing soybean seeds. Cysteine was nearly twofold higher in ATP sulfurylase overexpressing seeds than in the wild-type seeds. Similarly, sulfate was also higher in ATP sulfurylase overexpressing seeds. Several N-acetylated amino acids, such as N-acetyltryptophan, N-acetylglutamate, N-acetylglutamine, N-acetyl-3-methylhistidine and N-acetyl-1-methylhistidine were also increased in ATP sulfurylase overproducing seeds (Fig. 5). Overexpression of ATP sulfurylase had a significant effect on lipid metabolism. Several classes of lipids increased in ATP sulfurylase overexpressing seeds relative to the wild type control (Fig. 6). Many of these classes are components of membranes, such as phospholipids, sulfolipids, galactolipids, and sterols, but also included diacylglycerols and monoacylglycerols.

The levels of 26 metabolites involved in the amino acid super pathway was significantly lower (*p* < 0.05) in ATP sulfurylase overexpressing soybean seeds compared to control non-transgenic seeds (Supplemental Table 2). The concentrations of γ-glutamylalanine, γ-glutamylglutamine, γ-glutamylleucine and γ-glutamylvaline, which are related to glutathione metabolism, were noticeably lower in ATP sulfurylase overexpressing soybean seeds. Similarly, modest decreases in TCA cycle metabolites (i.e., citrate, tricarballyate, isocitrate and malate) were also observed in ATP sulfurylase overexpressing soybean seeds (Supplemental Table 2).

### Overexpression of ATP sulfurylase improves the sulfur amino acid content of seeds

Because the transgenic soybeans generated in this study exhibited elevated levels of ATP sulfurylase activity, we examine if the overexpression led to an increase in the sulfur amino acid content of the seeds. For this purpose, we quantified protein-bound amino acid content of four independent transgenic plants and compared with the non-transgenic soybean seeds. The amino acid content was determined with the use of high-pressure liquid chromatography (HPLC) (Table 1). The protein-bound cysteine content (expressed in mole %) in non-transgenic soybean seeds was 1.83% compared to 2.57%, 2.50%, 2.60% and 2.78% in the four independent transgenic soybean plants overexpressing ATP sulfurylase (Table 1). This represents 40.4%, 36.6%, 42.0%, and 51.9% increases in the cysteine content in these ATP sulfurylase overexpressing plants. Similarly, methionine content in non-transgenic soybean seeds was 1.46% compared to 1.71%, 1.69%, 1.68%, and 1.74% in the four transgenic soybean plants overexpressing ATP sulfurylase (Table 1). Arginine, valine and leucine content in all the four ATP sulfurylase overexpressing seeds were lower than in the wild-type seeds (Table 1).

## Discussion

Enzymes of the sulfur assimilation pathway are potential targets for improving nutrient content of important crops^1–6^. In this pathway, ATP sulfurylase catalyzes the formation of APS and inorganic pyrophosphate (PP_i_) from sulfate and ATP. The production of APS by ATP sulfurylase is the committed step in plant sulfur assimilation and is responsible for the generation of a high energy phosphosulfate bond that drives subsequent metabolic steps in the pathway^15–17^. Recent studies have targeted the overexpression of rate-limiting enzymes to accelerate the flux through the entire pathway. For example, serine acetyltransferase and OASS, two key cysteine biosynthesis enzymes, have been overexpressed in different plants including maize, lupin, potato, and rice leading to an increase in the cysteine and/or methionine content^8,9,31–33^. Some of these transgenic plants accumulated toxic intermediates resulting in growth abnormalities^34^. Earlier, we successfully overexpressed cytosolic isoform of OASS in transgenic soybean and observed a 58–74% increase in the protein-bound cysteine compared to wild-type plants^9^. Unfortunately, overexpression of OASS in transgenic soybeans caused growth reduction and impaired nodulation^35^. In the current study, constitutive overexpression of ATP sulfurylase under the control of CaMV promoter resulted in a 37–52% and 15–19% increase in the protein-bound cysteine and methionine content of transgenic seeds. Though the extent of increase the sulfur amino acid content is not as high as in the case of OASS overexpressed transgenic soybean plants, it was still accompanied by a slight growth reduction. Based on these observations, growth retardation observed in transgenic plants due to overexpression of sulfur assimilatory enzymes needs to be overcome to make this approach practicable. Previously, it was demonstrated that overexpression of APS reductase, an enzyme that catalyzes the conversion of APS to sulfite, also resulted in stunted plants; however, this negative effect was completely over come when an unregulated APS reductase was placed under the control of mesophyll-specific *PepC* or bundle sheath cell-specific *RbcS* promoter^14^. A similar approach may be required to overcome the negative effect of ATP sulfurylase overexpression in transgenic soybeans.

Metabolite profiling revealed significant changes in N-acetylated amino acids, which were increased in ATP sulfurylase overexpressing seeds relative to wild-type seeds. The reason for this increase is not clear. It seems likely that these compounds arise non-enzymatically, possibly by a reaction with acetyl-CoA. Such reactions are known to occur under relatively basic conditions within cells or in vitro^36^. It is possible that the ATP sulfurylase overexpressing seeds either contained higher levels of acetyl-CoA or that the internal pH or other conditions were more conducive for the reaction to occur. Another interesting aspect that was uncovered by our metabolite analyses relates to lipid metabolism. Several classes of lipids showed significant increase in ATP sulfurylase overexpressing seeds levels relative to the wild-type control seeds. Many of these lipids are components of membranes, such as phospholipids, sulfolipids, galactolipids, and sterols. Higher levels of membrane lipids imply that the transgenic seeds harbored more membrane structures than the control seeds. Increased diacylglycerides could result either from degradation of triacylglycerides (which are not measured here) or from new synthesis of phospholipid precursors. The increased amounts of lyso-phospholipids imply some lipolysis and membrane turnover is occurring^37^. The role of all these metabolite changes in transgenic soybean seeds and how it is related to altered flux in sulfur assimilatory pathway need further investigation.

Overexpression of ATP sulfurylase resulted in major changes in the protein profile of the soybean seeds. The two most abundant seed storage proteins of soybean are the 7S β-conglycinin and 11S glycinin^38,39^. Because of their abundance the nutritive value of soybean is mainly dependent on these two group of proteins. The 11S glycinin is relatively rich in sulfur amino acids when compared to the 7S β-conglycinin^40^. The β-conglycinin are glycoproteins and are composed of α′-, α-, and β-subunits. Interestingly the β-subunit of β-conglycinin is totally devoid of both methionine and cysteine^41^ and their abundance may lower the nutritive value of soybean seed proteins^42^. In our study, we found that overexpression of ATP sulfurylase resulted in drastic reduction in the accumulation of the β-subunit of β-conglycinin. Interestingly, the accumulation of this subunit is influenced by various factors including hormones, nodulation, sulfur, and nitrogen^42,43^. Nitrogen, sulfur deficiency and exogenous application of O-acetylserine, a cysteine precursor, to immature cotyledons promoted the accumulation of the β-subunit of β-conglycinin. In contrast, the accumulation of this subunit was drastically lowered when soybean plants were exposed to methionine or glutathione^44,45^. In our study, ATP sulfurylase overexpressing plants contained significantly higher amounts of cysteine and methionine. The increase in the methionine availability in transgenic seeds may be a contributing factor for the suppression of the β-subunit of β-conglycinin accumulation observed in our study. A recent study reported a 31.5% increase in the sulfur amino acid content in soybean lines that tend to accumulate low level of the β-subunit of β-conglycinin^46^. Transcriptome analysis of this soybean line revealed up regulation of genes involved in anabolism of cysteine, methionine and glutathione indicating a close relationship between sulfur amino acid content and the β-subunit of β-conglycinin accumulation^46^.

Previously, we reported transgenic soybean plants overexpressing a cytosolic OASS contained higher amounts of BBI^9^. Similarly, in this study we have demonstrated that overexpression of ATP sulfurylase also causes a dramatic increase in the accumulation of BBI. OASS, a member of the β-substituted alanine synthase enzyme family, is the last committed enzyme in the cysteine biosynthetic pathway, while ATP sulfurylase is the first enzyme in the sulfur assimilatory pathway. Overexpression of either of these two enzymes leads to elevated levels of cysteine. Soybean seeds responds to excess cysteine by promoting the accumulation of BBI, a protein that contains 14 cysteine residues^47,48^. The elevated levels of BBI in these transgenic plants, though significantly increases the overall sulfur-amino acid content of the seeds, may limit its utilization in the animal feed. Since protease inhibitors are major anti-nutritional factors preventing protein digestion, emphasis should be placed to not only reduce the inhibitory activity but also to maintain or elevate the sulfur-containing amino acid content of the seeds. An earlier study has shown that it is possible to generate soybean seeds with lower BBI activity by expressing inactive form of the inhibitor gene in transgenic soybeans^49^.

The relative abundance of BBI can be used as an indicator of the sulfur amino acid content of soybean seed proteins and can be exploited as an indirect measurement of cysteine content. Currently, HPLC is used to measure the cysteine and methionine content. However, this method is time-consuming, expensive, and not amenable for high-throughput screening. As an alternative approach, cysteine content of soybean seeds can be indirectly measured by ELISA using antibodies raised against BBI. This approach will enable high-throughput screening of thousands of soybean lines and enable identification of potential soybean cultivars with high sulfur amino acids.

To date, efforts to alter sulfur amino acid content in various plants, including maize, rice, and soybean, have resulted in increased cysteine and/or methionine levels, but also a range of other effects on plant metabolism, seed composition, and plant growth^8,9,13,14,31–33,35,50–53^. As shown here and in previous work^46^, increased expression of either sulfur assimilation (ATP sulfurylase) or cysteine biosynthesis (OASS) enzymes in soybean enhance sulfur-containing amino acid content but with rebalanced distribution of sulfur-rich seed proteins. Similar approaches that enhancement of sulfur metabolism in maize led to increases of methionine-rich zein seed proteins without negative effects on plant growth^13,14^, which highlights differences in nutrient metabolism and seed development in different crop plants. Plant growth relies on source-sink relationships, along with associated control mechanisms; however, these vary both by nutrient and by plant. Importantly, the plasticity of amino acid source-sink relationships is critical to allowing for compositional changes in seeds for survival of the next generation^52^. Yet, how targeted changes in plant metabolism, such as overexpressing a key enzyme like ATP sulfurylase in the sulfur assimilation pathway, alters metabolome and/or proteome plasticity remains to be understood in a predictive fashion.

## Materials and methods

### Plasmid construction

We previously reported the cloning and sequencing of Glyma10g38760a, a cDNA that codes for soybean ATP sulfurylase isoform 1^23^. The coding region of soybean ATP sulfurylase isoform 1 lacking the plastid localization sequence (GmATP SULFURYLASEΔ48) was amplified from the cDNA clone with appropriate primers^23^. To the amplified DNA, BamHI and NotI restriction sites were introduced for facilitation of cloning into an intermediate vector resulting in plasmid pATPS1. Following this step, the insert from the intermediate plasmid pATPS1 was digested with BamHI and XbaI and then cloned into the corresponding sites of pZ35S1, resulting in pZATPS1. The final plant transformation vector consisted of cauliflower mosaic virus 35S promoter (CaMV 35S), the ATP sulfurylase coding region, together with the cassette containing the CaMV 35S promoter, the bar-coding region and the 3′-region of the nopaline synthase gene. The soybean transformation vector pZATPS1 was transferred of into *Agrobacterium tumefaciens* (strain EHA105) by triparental mating.

### Production of transgenic soybean lines overexpressing ATP sulfurylase

Transgenic soybean plants were generated at the Plant Transformation Core Facility at the University of Missouri, Columbia. *Agrobacterium*-cotyledonary node transformation was used to transform Soybean cv. Maverick and regenerated transgenic soybean plants were screened for tolerance to herbicide Liberty by a leaf-painting assay. DNA from plants showing resistance to the herbicide was used for PCR to verify the integration of the introduced ATP sulfurylase. The overexpression of ATP sulfurylase from four independent transgenic soybean lines was verified by immuno-blot analysis and by measuring ATP sulfurylase activity, as detailed below.

### Plant growth

All the four independent transgenic soybean lines (ATPS1, ATPS2, ATPS3, and ATPS4) overexpressing ATP sulfurylase were advanced to T4 generation at the Sears Plant Growth Facility, University of Missouri, Columbia, in 2-gallon pots containing PROMIX (PremierHorticulture, Quebec, Canada). Greenhouse settings were 16-h daylength and 30/18 °C day/night temperatures. During the growing season the plants were fertilized twice with Osmocote Plus. After obtaining regulatory permit from Animal and Plant Health Inspection Service, Biotechnology Regulatory Services (APHIS-BRS), U.S. Department of Agriculture, homozygous ATP sulfurylase overexpressing transgenic plants representing a single transgenic event (ATPS1) were also grown in the field at Bradford Research and Extension Center, Columbia, Missouri in 2017 and 2018. Cultural practices were typical of those used for soybean production in the Midwest USA.

### Determination of oil and protein content

Mature seeds harvested from field-grown homozygous transgenic soybean plants and non-transgenic control plants were used for the determination of protein and oil content. Near-infrared reflectance spectroscopy (Tecator AB, Hoganas, Sweden) was used to quantify oil content. Protein content was quantified using the LECO Model FP-428 nitrogen analyzer.

### One-dimensional and two-dimensional SDS-PAGE

For extraction of total proteins 10 mg of finely ground dry soybean seed powder was placed into a 2 ml Eppendorf tube containing with 1 ml of 1X SDS sample buffer (0.06 M Tris–HCl, pH 6.8, 2% SDS (w/v), 10% glycerol (v/v), and 5% 2-mercaptoethanol (v/v). In the case of developing seeds, 100 mg of seed tissue was used for total protein extraction. The contents of the tubes were vigorously agitated on a vortex followed by centrifugation at 16,100 × *g* for 10 min at room temperature. The resulting supernatant was transferred to a new plastic tubes and placed in a boiling water bath for 5 min. A small aliquot (10 µL) were routinely used to separate the total proteins by electrophoresis. Protein separation on 1-D gels (13% SDS-PAGE), was at a constant current of 20 mA/gel in a Mini250 gel apparatus (GE Healthcare). Separated proteins were visualized by staining the gels in Coomassie R-250.

2-D gel electrophoresis was carried out as previously described^54^. After electrophoresis, gels were immediately removed and fixed in 5:4:1 (methanol:water:acetic acid) solution for 1 h, followed by two brief rinses in ultrapure water, then stained in a Coomassie G-250 solution for overnight.

### Image acquisition and analysis

1-D and 2-D Coomassie-stained gels were destained with multiple changes of water to remove background. Gels were scanned using an Epson V700 Perfection scanner controlled through Adobe Photoshop. Delta2D software (Decodon GmbH, Greifswald, Germany) was used for differential image analysis and provided the % protein spot volume data for comparison. Delta2D default parameters were set to maximize spot detection using global image warping and exact spot matching within and between groups (Group 1, Maverick, n = 3; Group 2, ATPS, n = 3). Percent spot volume values were determined for a select group of proteins and error is reported as the standard error of the mean.

### Immunoblot analysis

SDS-PAGE separated proteins were electrophoretically transferred to nitrocellulose membranes as previously described^54^. Antibodies raised against soybean ATP sulfurylase^23^, soybean OASS^55^, and soybean Bowman–Birk protease inhibitor (BBI)^9^, were used for immunoblot analysis at 1:10,000 dilution. The β-subunit of β-conglycinin antibody^56^ was used at 1:100,000 dilution. Following the incubation with the antibodies, the nitrocellulose membranes were washed in three changes of TBST (0.01 M Tris–HCl, pH 7.5, 0.5 M NaCl and 0.2% Tween 20) and incubated with goat anti-rabbit IgG-horseradish peroxidase conjugate (Bio-Rad) that had been diluted at 1:20,000. Immunoreactive proteins were detected utilizing a Super Signal West Pico chemiluminescence kit (Pierce).

In order to localize the ATPS activity in soybean plants, freshly harvested leaf material was subjected cell fractionation following established protocol^57^. Two grams of fresh leaf material harvested from Maverick and ATPS overexpressing soybean plants were individually homogenized with 10 ml of 50 mM HEPES–KOH, pH 7.5, 250 mM sorbitol, 50 mM KOAc, 2 mM Mg(OAc)2, 1 mM EDTA, 1 mM EGTA, and 1 mM DTT and protease inhibitor cocktail. The slurry was subjected to multiple centrifugation steps in order to obtain crude chroplastic, endoplasmic reticulum and cytoplasmic protein fractions^57^. Equal amount of proteins (30 μg) representing the different subcellular fractions were resolved by SDS-PAGE followed by western blot analysis using ATPS antibody as described above.

### ATP sulfurylase activity assay

Protein extracts from non-transgenic control and transgenic seeds were used for measuring ATP sulfurylase activity. ATP sulfurylase activity in developing soybean seeds was measured in the reverse direction (adenosine 5′-phosphosulfate + PP_i_ → adenosine 5′-triphosphate + sulfate) using the continuous spectrophotometric rate determination method^58,59^, by coupling adenosine 5′-triphosphate production with hexokinase (adenosine 5′-triphosphate + D-glucose → D-glucose 6-phosphate + adenosine 5′-diphosphate) and glucose-6-phosphate dehydrogenase (D-glucose 6-phosphate + NADP → 6-phospho-D-gluconate + NADPH). Enzyme assays (1 mL) contained 0.1 M Tris–Cl, pH 8.0, 0.0025 M magnesium acetate, 0.035 M D-glucose, 0.001 M adenosine 5′-phosphosulfate, 4 U hexokinase, 2 U glucose 6-PO_4_ dehydrogenase, 0.001 M DTT, 0.001 M NADP^+^, and 0.0035 M pyrophosphate. Activity is expressed as nmol of NADPH produced per minute per mg at pH 8.0 at 30 °C in the coupled assay.

### Amino acid analysis

Dry seeds from four independent transgenic events (ATPS1, ATPS2, ATPS3, ATPS4) and a wild-type soybean line (Maverick) were ground to a fine powder and subjected to hydrolyzed amino acid analysis (Proteomics & Metabolomics Facility at the Center for Biotechnology/ University of Nebraska – Lincoln). Amino acids were quantified using the Waters AccQ-Tag Ultra Kit on an Acquity UPLC system. In this procedure, Cys and Met are quantified as CyA (cysteic acid) and MetS0_2_ (methionine sulfoxide), Gln and Asn are converted to Glu and Asp, respectively while Trp is destroyed during hydrolysis. Three biological replicates were used for amino acid measurements. Statistical analysis was performed using JMP 14.0.0 statistical software. Mean amino acid (% mole) were compared via ANOVA and for tests which showed significant differences between wild-type and transgenic seeds means were compared through Tukey’s HSD; genotypes which are not significantly different are indicated by overlapping letters.

### Metabolite analysis by LC–MS and GC–MS

Global metabolic profiles were determined using the Metabolon HD4 global metabolomics platform (Metabolon Inc., Research Triangle Park, North Carolina). The procedure used for extraction of metabolites and analysis on LC–MS and GC–MS platforms has been previously described^35^. Metabolite analysis was performed with 4 biological replicates and all statistical analyses were carried out using a confidence threshold of α = 0.05. ANOVA and t-tests were performed in JMP software using the normalized data obtained from the LC–MS and GC–MS platforms. Statistical analyses were performed using JMP Version 11 software (SAS Institute Inc., Cary, NC, USA) and statistical computing and graphic was carried out with “R” (https://cran.r-project.org/).

## Supplementary information

Supplementary Information.
