# Supplementary material for: Overexpression of ATP sulfurylase improves the sulfur amino acid content, enhances the accumulation of Bowman–Birk protease inhibitor and suppresses the accumulation of the β-subunit of β-conglycinin in soybean seeds
Source: Sci Rep. 2020 Sep 14;10:14989. doi: 10.1038/s41598-020-72134-z (PMC7490426; doi:10.1038/s41598-020-72134-z)
Supplement: Supplementary file 1 — Supplementary Information. [file 41598_2020_72134_MOESM1_ESM.docx]

**Supplementary Information**

**Overexpression of ATP sulfurylase improves the sulfur amino acid content, enhances the accumulation of Bowman-Birk protease inhibitor and suppresses the accumulation of the β-subunit of β-conglycinin in soybean seeds**

# Won-Seok Kim^1^, Jeong Sun-Hyung^2^, Nathan W. Oehrle^2^, Joseph M. Jez^3^, and Hari B. Krishnan^1,2,*^

# ^1^Plant Science Division, University of Missouri, Columbia, MO 65211

# ^2^Plant Genetics Research, USDA-Agricultural Research Service, Columbia, MO 65211

# ^3^Department of Biology, Washington University in St. Louis, St. Louis, MO 63130

# * Corresponding author’s e-mail: [hari.b.krishnan@usda.gov](mailto:hari.b.krishnan@usda.gov)

#
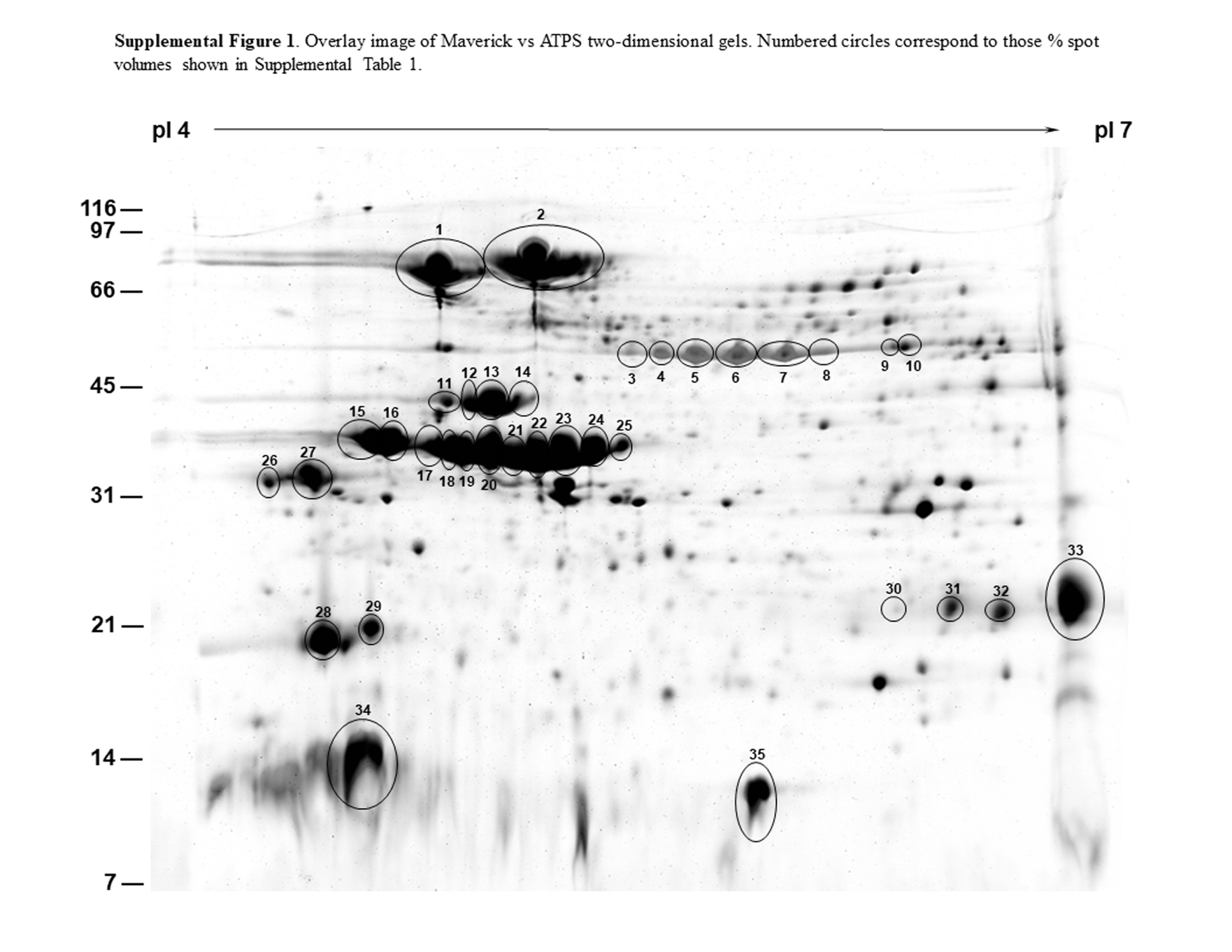


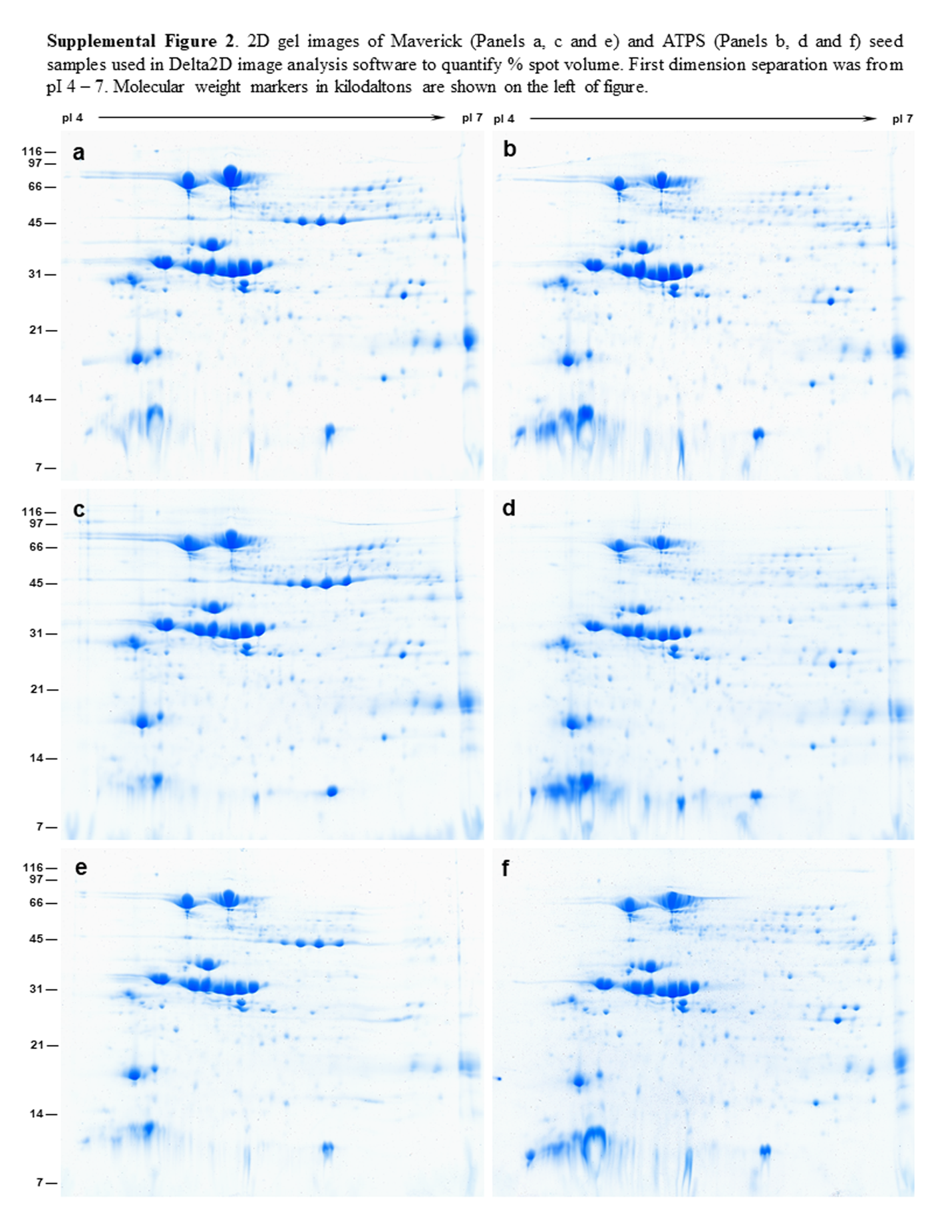


**Supplemental Table 1**. Two-dimensional gel, Delta2D image analysis software spot quantification results between Maverick and ATPS seed proteins. Percent spot volumes of the main seed storage proteins, Kunitz trypsin inhibitor and Bowman-Birk protease inhibitor are reported as the average between three separate protein separations and spot quantifications, along with the standard error (n=3). Spot #’s correspond to those circled protein spots in Supplemental Figure 1, which have been identified in our previous publication (Figure 2A [28]).

| **Spot #** | **Spot # in**  **Fig.2A [28]** | **Protein Identification [28]** | **% Spot Volume**  **Maverick**  **Avg. (n=3)** | **S.E.** | **% Spot Volume**  **ATPS**  **Avg. (n=3)** | **S.E.** |
| --- | --- | --- | --- | --- | --- | --- |
| 1 | 1 | β-conglycinin (α-subunit) | 5.155 | 0.645 | 3.872 | 0.283 |
| 2 | 2 | β-conglycinin (αʹ-subunit) | 7.589 | 0.677 | 5.893 | 0.507 |
| 3 | 9 | β-conglycinin (β-chain, A) | 0.393 | 0.142 | 0.086 | 0.009 |
| 4 | 10 | β-conglycinin (β-chain, A) | 0.876 | 0.201 | 0.109 | 0.016 |
| 5 | 11 | β-conglycinin (β-chain, A) | 1.548 | 0.244 | 0.117 | 0.017 |
| 6 | 12 | β-conglycinin (β-subunit) | 2.127 | 0.463 | 0.221 | 0.032 |
| 7 | 13 | β-conglycinin (β-chain, A) | 1.477 | 0.274 | 0.135 | 0.031 |
| 8 | 14 | β-conglycinin (β-subunit) | 0.518 | 0.048 | 0.136 | 0.010 |
| 9 | 16 | β-conglycinin (α-subunit) | 0.169 | 0.021 | 0.150 | 0.013 |
| 10 | 17 | β-conglycinin (α-subunit) | 0.233 | 0.058 | 0.206 | 0.057 |
| 11 | 18 | Glycinin (A3B4) | 0.538 | 0.032 | 0.678 | 0.011 |
| 12 | 19 | Glycinin (A3B4) | 0.829 | 0.102 | 0.658 | 0.082 |
| 13 | 20 | Glycinin (A3B4) | 2.234 | 0.152 | 1.943 | 0.248 |
| 14 | 21 | Glycinin (A3B4) | 0.642 | 0.080 | 0.434 | 0.072 |
| 15 | 22 | Glycinin (partial) | 1.983 | 0.089 | 1.677 | 0.045 |
| 16 | 23 | Glycinin (partial) | 2.023 | 0.242 | 1.858 | 0.209 |
| 17 | 24 | Glycinin (A2B1a) | 1.113 | 0.074 | 1.018 | 0.064 |
| 18 | 25 | Glycinin (A2B1a) | 1.315 | 0.136 | 1.258 | 0.150 |
| 19 | 26 | Glycinin (A2B1a) | 1.287 | 0.156 | 1.320 | 0.191 |
| 20 | 27 | Proglycinin (A1ab1b) | 2.430 | 0.269 | 2.603 | 0.290 |
| 21 | 28 | Proglycinin (A1ab1b) | 1.709 | 0.190 | 1.869 | 0.220 |
| 22 | 29 | Glycinin (A1aB1b) precursor | 2.281 | 0.270 | 2.483 | 0.361 |
| 23 | 30 | Glycinin (A1aB1b) precursor | 2.798 | 0.325 | 2.697 | 0.286 |
| 24 | 31 | Proglycinin (A1ab1b) | 2.298 | 0.204 | 1.823 | 0.251 |
| 25 | 32 | Glycinin (A1aB1b) precursor | 0.571 | 0.080 | 0.433 | 0.106 |
| 26 | 36 | Glycinin (partial) | 0.637 | 0.054 | 0.614 | 0.099 |
| 27 | 37 | Glycinin (partial) | 2.804 | 0.347 | 2.496 | 0.189 |
| 28 | 42 | Kunitz trypsin inhibitor, chain A | 3.482 | 0.201 | 3.774 | 0.410 |
| 29 | 43 | Kunitz trypsin inhibitor, precursor | 0.970 | 0.012 | 0.972 | 0.039 |
| 30 | 45 | Glycinin (A1aB1b) precursor | 0.092 | 0.012 | 0.066 | 0.014 |
| 31 | 46 | Glycinin (A2B1a) precursor | 0.842 | 0.042 | 0.663 | 0.038 |
| 32 | 47 | Glycinin (A1aB1b) precursor | 1.097 | 0.025 | 0.869 | 0.037 |
| 33 | 48 | Glycinin (A1aB1b) precursor | 3.422 | 0.117 | 2.649 | 0.763 |
| 34 | 50 | Bowman-Birk protease inhibitor | 2.803 | 0.448 | 5.343 | 0.749 |
| 35 | 51 | Glycinin (A5A4B3) precursor | 2.023 | 0.069 | 2.145 | 0.061 |

# Supplemental Table 2. Heat map of statistically significant biochemicals profiled in this study.

# 
